# Occurrence of organic pollutants in the River Itchen and River Test—two chalk streams in Southern England, UK

**DOI:** 10.1007/s11356-022-23476-w

**Published:** 2022-10-07

**Authors:** Rosamund F. A. Robinson, Graham A. Mills, Anthony Gravell, Melanie Schumacher, Gary R. Fones

**Affiliations:** 1 School of the Environment, Geography and Geosciences, University of Portsmouth, Burnaby Road, Portsmouth, PO1 3QL UK; 2 School of Pharmacy and Biomedical Sciences, University of Portsmouth, White Swan Road, Portsmouth, PO1 2DT UK; 3 Natural Resources Wales, Faraday Building, Swansea University, Singleton Campus, Swansea, SA2 8PP UK

**Keywords:** Chalk streams, Suspect screening, Organic chemicals, Polar pesticides, Pharmaceuticals, Surface water

## Abstract

The River Itchen and River Test, two chalk streams in Southern England, are sites of special scientific interest. These ecosystems face a number of environmental pressures from anthropogenic inputs of organic pollutants. Hence, we investigated the occurrence of these chemicals within the two catchments. Spot water samples (1 L) were collected at nineteen sites along the catchment on two occasions (March and June 2019). Samples were extracted (HLB-L sorbent disks) and analysed using high-resolution liquid chromatography-quadrupole-time-of-flight mass spectrometry and gas chromatography-mass spectrometry. Compounds were identified against commercially available databases. Using this approach, we found 115 pharmaceutical and personal care products, 81 plant protection products and 35 industrial chemicals. This complex mixture of pollutants covered a range of physico-chemical properties and included priority substances in the EU Water Framework Directive or currently on the third Watch List. Both rivers had similar chemical profiles for both months. Herbicides and fungicides were dominant in the spring, whereas insecticides occurred more frequently in the summer. Point discharges from wastewater treatment plants were the main source of pharmaceutical and personal care products. Agricultural activities were the main contributor to the presence of plant protection products. The impact of these organic chemicals on the ecology, particularly on macroinvertebrate biodiversity, is unknown and warrants further investigation. Our suspect screening approach could guide future toxicological investigations to assess the environmental impacts of these diverse chemicals.

**Supplementary Information:**

The online version contains supplementary material available at 10.1007/s11356-022-23476-w.

## Introduction

A chalk river arises via springs from a chalk aquifer where rainwater has percolated through the ground and stored in the porous rock. The groundwater level in the aquifer is naturally cyclical increasing when recharged by rainfall and decreasing by drainage via springs. The water level is also reduced by groundwater and river water extraction (Cao et al. 2020). England has 85% of the world’s chalk streams, totalling 161 streams mainly located in the south of the country. The other 15% are found in northern and western France (Maréchal and Rouillard 2020). The water has a high alkalinity (> 50 mg L^−1^ CaCO_3_) with a pH ranging from 7.4 to 8 and a small temperature range between 5 and 17 °C. Chalk rivers have a stable hydrological regime and supports diverse communities of aquatic plants, invertebrates, insects and fish. Often groundwater abstraction can reduce the river flow to low levels; this can have an impact on aquatic communities. In order to negate this effect, wastewater from wastewater treatment plants (WWTPs) is discharged to the rivers. Chalk streams can be polluted by a wide range of organic chemicals (OCs). These include pharmaceuticals and personal care products (PPCPs) (Daughton and Ternes 1999), plant protection products (PPPs) (Barco-Bonilla et al. 2010) and industrial chemicals (IND) (Bunting et al. 2021).

Human and animal pharmaceutical products are designed to act within the body at low doses. Once ingested, they are excreted either as the parent drug or as a range of metabolites (Petrie et al. 2015). Pharmaceuticals have different therapeutic effects and are classified depending on the system in the body being treated (e.g. central nervous, circulatory and respiratory). Personal care products used to improve the quality of life include anti-perspirants, detergents, soaps and sunscreens (Kasprzyk-Hordern et al. 2008). PPCPs enter the aquatic environment from a variety of sources. Primary routes include discharges from WWTPs and the manufacture of these products (Al Aukidy et al. 2012). Additionally, during periods of heavy rainfall, combined sewer overflows (CSOs) can discharge untreated wastewaters directly into rivers (Munro et al. 2019). Secondary routes include leachate from animal slurry (Boxall et al. 2004) and landfills sites (Bound and Voulvoulis 2005) and run off from fields treated with slurry from WWTPs (Pederson et al. 2005).

PPPs are chemicals used for controlling agricultural pests and diseases. They are divided into different groups depending on their use (e.g. fungicides, herbicides and insecticides), and for some, the active substance may also be registered as a veterinary medicine. Fungicides are applied to crops and additionally can be used externally on animals. Herbicides are used for pre-emergence of weeds and regular control of weeds. Insecticides are used as seed treatments, externally on animals and applied to crops. Larvicides interrupt the development of larvae and applied to crops, in greenhouses and onto manure. Finally plant growth regulators are used to modify plant growth by acting on the plant’s hormonal processes (Rademacher 2015). The primary pathway for PPPs to enter surface waters is run-off from treated fields (Cruzeiro et al. 2016). Additional routes include airborne during spray application (Bonmatin et al. 2014) and through leachate to the aquifer (Marsala et al. 2020).

IND are used in manufacturing processes and include adhesives, dyes, flame retardants, plasticisers and solvents. These chemicals may be discharged into surface waters via WWTPs or by the manufacturing industry. Products of combustion (e.g. polyaromatic hydrocarbons (PAHs)) are also included in this classification.

Chemical pollution in surface waters may pose a significant risk to the aquatic environment. Aquatic organisms are captive to the continual exposure of OCs entering surface waters (Daughton and Ternes 1999). Research has shown behavioural and developmental effects from OCs on fish (Sehonova et al. 2018), shellfish (Almeida et al. 2017), algae and invertebrates (Oskarsson et al. 2012). In addition, any OCs that sorb onto sediments may be disturbed by high water flows and benthic dwelling organisms and become re-suspended in the water column (Nicolaus et al. 2015).

Many OCs that are present in the aquatic environment are subject to legislation. For example, within the European Union, this is covered by the Water Framework Directive (WFD) 2008/105/EC, (European Commission 2008) and its related daughter directives. Similar legislation is in operation in other jurisdictions. Under the WFD, a Watch List (WL) of substances to be monitored and quantified is specified and reviewed every 2 years (Carvalho et al. 2015). For many OCs, environmental quality standards (EQSs) have been established (Pietrzak et al. 2019).

Monitoring of OCs in water is important to understand their occurrence, sources and fate. This is generally undertaken by the collection of low volume (1–2 L) spot samples of water followed by instrumental analysis (gas chromatography-mass spectrometry (GC–MS) or liquid chromatography-mass spectrometry (LC–MS)) in the laboratory. Traditionally this has been targeted analysis for the measurement of a pre-defined list of analytes. This limited approach can miss many substances that can have an ecological impact. Hence, more recently alternative approaches have been used such as screening which employs suspect (Taylor et al. 2020) and non-target analysis (Overdahl et al. 2021).

This study investigated the presence of a wide range of OCs in two chalk streams (River Itchen and River Test) in the south of England, UK. These rivers have a similar ecology and have been designated as Sites of Special Scientific Interest (SSSI). In addition, the River Itchen has been designated as an international Special Area of Conservation (SAC) (https://sac.jncc.gov.uk/site/UK0012599). The rivers support a diversity of wildlife and commercial activities such as trout and salmon fishing, watercress production and fish farming. Agricultural practices in the two areas are similar. However, there are differences in other major anthropogenic inputs (e.g. number of WWTPs) within the two catchments. Spot water samples were collected at various points along the rivers in both spring (March) and summer (June) 2019. These dates were selected to coincide with the expected application of differing PPPs within the catchment. Samples were pre-concentrated using solid-phase extraction (SPE) disks and the extracts analysed qualitatively using two suspect screening approaches (GC–MS and LC–MS) to identify organic contaminants without a priori knowledge of the pollutants present.

There have been limited studies investigating the range of organic pollutants in chalk streams in the UK. The aim of this suspect screening study was to elucidate the occurrence and distribution of these pollutants. Chemical profiling information from the study will be important for assessing the potential long-term impact that these substances may have on the biota of chalk streams. This qualitative information may also guide future targeted (quantitative) monitoring campaigns and help direct mitigation and risk assessment strategies within the catchment.

## Materials and methods

### Study area

**Fig. 1:**
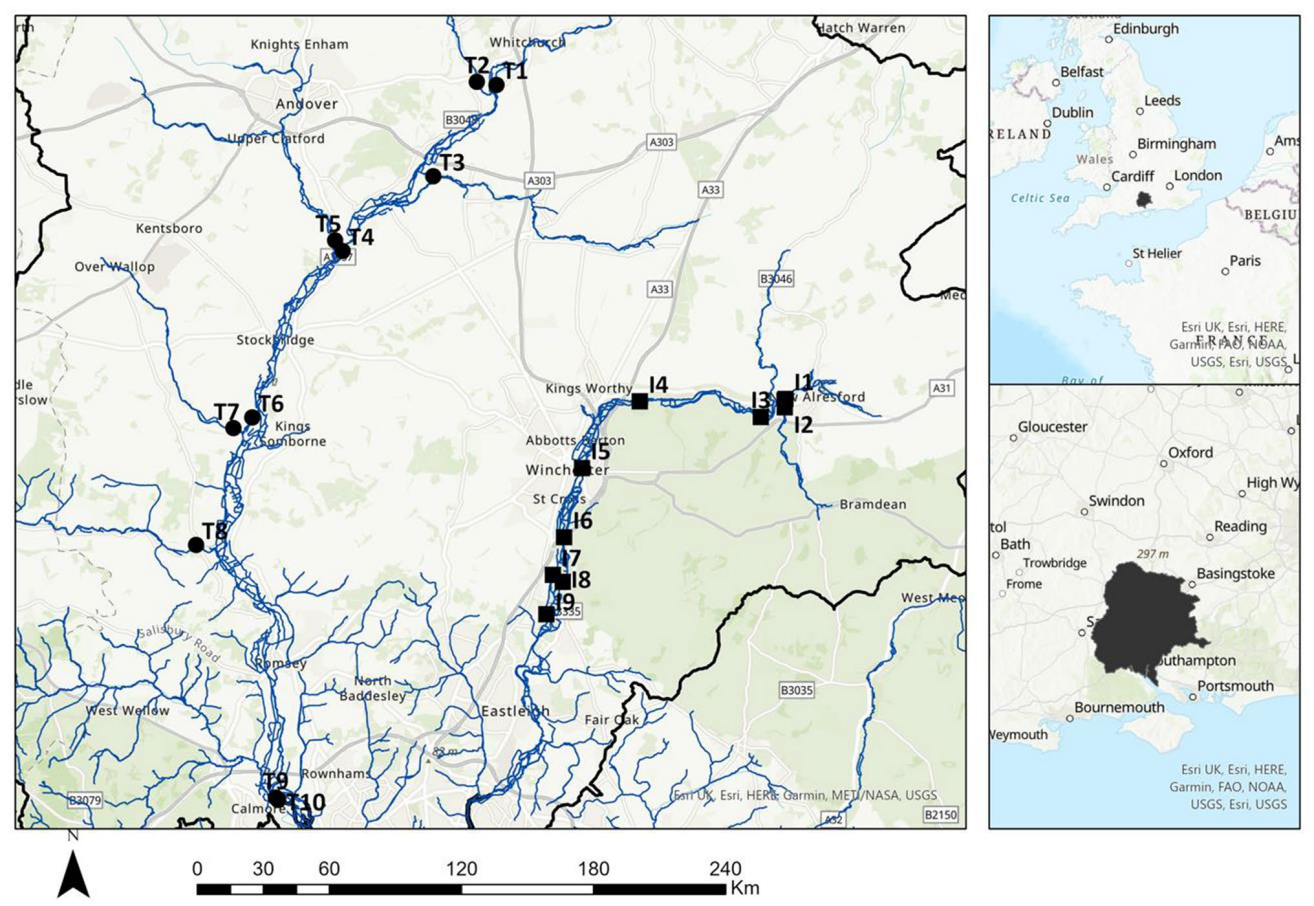
Map of the nineteen sampling site locations on the River Itchen (black-shaded square) and River Test (black-shaded circle) catchment. The two right hand panel insets show the location of the study site within Europe and within Southern England. Numbering of sites in each river begins at the uppermost site and continues sequentially downstream. Sampling sites for the River Itchen (I1-I9) and River Test (T1-T8) were in the chalk area of the catchment. Sampling sites for the River Test (T9 and T10) were in the southern part of the catchment where the riverbed is dominated by clay, silt and sand sediments (Allen 2017). Some features of this map are based on spatial data licences from the UK Centre for Ecology & Hydrology, UKCEH, and contain Ordnance Survey data crown copyright and database right 2022 (Moore et al. 1994). Crown copyright and database rights 2021 OS 100,025,252. Further details on the sampling sites are given in the electronic supplementary material (Figs. S1 and S2 and Tables S1 and S2)

Monitoring was undertaken at nineteen locations within the River Itchen (ten sites) and River Test (nine sites) catchments (Fig. 1).

The study area is in the county of Hampshire (UK) where the permeable chalk aquifer (Allen 2017) in the north of the county is the source of the rivers Test and Itchen. The total catchment area is 1760 km^2^ and is primarily rural with 62% of the land used for farming. Arable faming such as wheat, barley, oats and oil seed rape accounts for 80%. In addition, there are areas of broadleaved, mixed and coniferous woodland, unimproved grassland and water meadows. Small areas are used for mixed farming including livestock. Major urban areas in the northern part are Andover, Romsey and Winchester; in contrast the southern part of the catchment is dominated by clay, giving different stream characteristics and habits for different aquatic organisms; the major urban areas in the south are Southampton, Totton and Eastleigh.

The River Test rises from the Chalk aquifer near Ashe near Basingstoke and flows southwards for approximately forty miles being joined by the Bourne Rivulet, the rivers Dever and Anton, then Wallop Brook, the river Dun and finally to Southampton Water. The River Itchen rises from chalk springs above Alresford and is fed by three main tributaries, Candover Brook, Cheriton Stream and the River Alre. Throughout much of its twenty-eight-mile length, it is divided into two or more channels parallel to each other and including the Itchen Navigation. The river discharges into Southampton Water. Both rivers support aquaculture such as watercress production, fish farming and leisure activities including trout and salmon fishing. In the catchment, there are domestic (7%) and business properties (14%) not connected to the sewerage system and rely on septic tanks and package treatment plants for their waste disposal. There are 30 municipal WWTPs in the two catchments (5 on the River Itchen and 25 on the River Test) (Table S3), with 30% discharging directly into the strata (Southern Water 2020).

Rainfall data for the sampling area was collected from the Met Office Hadley Centre observations datasets for SE England (https://www.metoffice.gov.uk/hadobs/hadukp) (Alexander & Jones 2001). Flow rate information was collected from gauging station data from the UK National River Flow Archive website (https://nrfa.ceh.ac.uk/data/search).

### Chemicals, glassware and reagents

Solvents were of HPLC-grade or better (Thermo Fisher Scientific, Loughborough, Leicestershire, UK). Ultra-pure water (UPW) (> 18.0 MΩ cm at 25 °C) was made using a Milli-Q® purification system (Merck, Burlington, USA) and was used in all laboratory procedures. Formic acid was from Sigma-Aldrich (Dorset, UK). Glassware was soaked overnight in a 5% Decon 90 solution (Decon Laboratories Ltd, Hove, UK) and rinsed with UPW then methanol (MeOH) before use.

### Sample collection and extraction

Spot water samples (2 × 1 L) were collected into green glass screw top bottles with no preservative added. Samples were collected on the 7th March and 20th June 2019 from the nineteen sites. Sampling was undertaken in March and June when it was expected that there would be higher usage of fungicides, herbicides and pesticides within the catchments. Sampling locations were selected to obtain an overview of the different inputs into the catchment including the main channel and tributaries. Further details of the sampling sites are given in Table S1 in the Electronic Supplementary Material (ESM). Samples were extracted (within 24 h of collection) using a Horizon Atlantic® HLB-L disk (Biotage, Sweden). This is a hydrophilic-lipophilic balanced sorbent designed to sequester polar and semi-polar analytes with a wide range of log *K*_*ow*_ (Taylor et al. 2020).

### Preparation of HLB-L disk

Each disk was soaked overnight in MeOH and then conditioned in an extraction manifold under gentle vacuum with MeOH (50 mL) followed by water (100 mL), ensuring the disk does not dry out (Taylor et al. 2020).

### Extraction of water samples for LC/MS analysis

Each sample bottle was inverted to ensure re-suspension of particles. Water samples (1 L) were extracted on the HLB-L disk using the extraction manifold under gentle vacuum. Disks were dried (24 h) in a laminar flow hood. Analytes were extracted using the extraction manifold and eluted under gravity with MeOH (40 mL) into a glass screw top vial (60 mL). Water (1 mL) was added to each vial as an analyte retainer. The eluent was evaporated to ~ 0.5 mL using a Genevac EZ-2 centrifugal rotary evaporator (Genevac Ltd. Ipswich, UK) set at 40 °C. Extracts were transferred to 2-mL silanized vials (Agilent, Santa Clara, USA), adjusted to 1 mL with MeOH and the weight recorded. Samples were stored at − 18 °C prior to instrument analysis by LC/MS. Three conditioned disks served as laboratory blanks. These were solvent extracted as above but without the addition of a water sample.

### Extraction of water samples for GC/MS analysis

Water samples (1 L) were extracted on HLB-L disks as above and spiked with an internal standard solution containing D_10_-phenanthrene. The extracts were dried by loading onto high-purity anhydrous sodium sulphate cartridges (15 g) (Agilent, Santa Clara, USA) and then eluted with n-hexane (20 mL). The eluents were evaporated as above to a final volume of 0.5 mL. Samples were stored at − 18 °C prior to instrument analysis by GC/MS. Three conditioned disks served as laboratory blanks. These were solvent extracted as above but without the addition of a water sample.

### Instrumental analysis

#### LC/MS

The analytical method used was liquid chromatography coupled with time-of-flight mass spectrometry (LC-Q-TOF) as described by Taylor et al. (2020). Briefly extracts were separated using a Dionex Ultimate 3000 UHPLC system (Thermo Fisher Scientific, Bremen, Germany) connected to a Bruker Maxis Impact II electrospray high-resolution time-of-flight tandem mass spectrometer (Q-TOF–MS) (Bruker Daltonics, Bremen, Germany). The resolution of the instrument was 30,000 at m/z 150–200. HyStar software (rev. 3.2) and Target Analysis for Screening and Quantification (TASQ®) 1.4 software (Bruker Daltonics, Bremen, Germany) were used for data acquisition and interpretation, respectively. Suspect compounds (~ 2500 substances included in the PesticideScreener™ 2.1 and ToxScreener™ 2.1 libraries) were identified in the extracts based on the retention time, mass accuracy, isotopic pattern and diagnostic MS/MS fragments.

#### GC/MS

Extracts were analysed on an Agilent 6890–5975 GC/MS system. Analytes were separated on a HP-5MS ultra inert capillary GC column (30.0-m length, 0.25-mm diameter, 0.25-µm film thickness) operated in constant pressure mode. The carrier gas pressure was adjusted against D_10_-phenanthrene, to give a retention time of 17.857 min.

The oven temperature programme was initial temp 40 °C (2.0 min) then 10 °C min^−1^ to 300 °C (8.0 min) and total run time 36.0 min. The interface temperature was maintained at 300 °C. The programmable injector was operated in splitless mode (initial temp 20 °C (0.05 min) then ramped at 720 °C min^−1^ to 300 °C. Analytes were identified using Agilent deconvolution reporting software (DRS) in conjunction with a hazardous chemical database containing the mass spectra of approximately 1000 pesticides, solvents, endocrine disruptors and related compounds. Database entries included retention times from the D_10_-phenanthrene locked method. A match in retention time and spectral ions and ion ratios provided a positive compound identification.

### Classification of organic chemicals

Data from the LC/MS and GC/MS analysis were categorised into three different classes, namely, PPCPs, PPPs and IND. Each of the classes was then subdivided and coded according to compound use. PPCPs were categorised (https://www.nhs.uk/medicines/) as prescription only medication (POM) and can only be purchased from a pharmacy (P) or purchased over the counter (general sales list, GSL). Where a PPCP had more than one use, this was reflected in the code, and metabolites were allocated the letter “M” plus the same code as the parent drug. Other categories included designer drugs (DD) and ingredients (ING). Substances subject to the Misuse of Drugs Act 1971 (MDA) and the Misuse of Drugs Regulations 2001 (MDR) are collectively identified as Misuse of Drugs (MoD) and annotated as class A, B or C.

PPPs were classified (http://sitem.herts.ac.uk/aeru/ppdb/en/index.htm) as herbicides (herb), fungicides (fung), insecticides/biocides (ins), larvicides (larv) and plant growth regulators (PGR) along with whether the pesticide had been authorised or not for use in the EU and if it is classified as a priority substance (PS) or appears on the WL of the WFD (European Commission 2008, 2013, 2020). IND were classified as industrial compounds according to the description given of their use on the EU REACH information cards (https://echa.europa.eu/information-on-chemicals/registered-substances). In addition, any substance found required to be monitored by the EU WFD (Environment Quality Standards and Watch Lists) (European Commission 2008, 2013, 2020) was also identified. Where a compound was positively identified by both GC/MS and LC/MS, it was recorded as just one data entry (see Tables 1 and 2).

**Table 1:** List of pharmaceuticals and personal care products (together with Chemical Abstracts number [CAS] Log *K*_*ow*_ and their Misuse of Drugs [MoD] class) detected along with their frequency (%) in extracts from the spot water samples collected from the River Itchen and Test in March and June 2019. Code: *AL&PK* Alzheimer’s and Parkinson’s disease, *ANHIS* antihistamines, *ANSTH/PYSTIM* anaesthetic and psychiatric stimulant, *ANSTH* anaesthetic, *ANTB* antibiotic, *ANTD* antidepressant, *BB* beta blocker, *BLAD* bladder, *BT* blood thinning, *CAN* cancer, *CHBP* heart and circulation, *CHOL* cholesterol, *CHOLIN* cholinergic, *CMED* cough medicine, *CNS STIM* central nervous system stimulant, *DIAB* diabetes, *DIUR* diuretic, *EPD* epilepsy, *FLU* influenza, *FUNG* fungal treatment, *HOR* hormone, *LAX* laxative, *M* metabolite, *MALD* malaria, *MRELX* muscle relaxant, *NAU* nausea, *NSAID* nonsteroidal anti-inflammatory drug, *OPAN* opioid drug, *PAR* paraben, *PERS* personal care, *PR* pain relief, *PYSM* psychotic drug, *PYSTIM* psychoactive stimulant, *RETRO* retrovirus, *SED/T* sedative, *SKIN* skin diseases, *ULC* ulcer, *UV* ultra violet filter. Category: *POM* prescription-only medication, *P* only available from a pharmacy, *GSL* general sales list (over the counter), *DD* designer drug, *ING* ingredient. Analytical detection: GC/MS = G; LC/MS = L. River detection: River Itchen = I; River Test = T; B = both rivers. WL = Watch List

Code	Compound	CAS	Log *K*_*ow*_	Category	MoD	G/L	River detected in March	River detected in June	Detection (%) in all samples (*n* = 38)
AL&PK/FLU	Amantadine	768–94-5	2.44	POM		L	T		3
ANHIS	Azacyclonol	115–46-8	2.9	POM		L		T	3
	Cetirizine	83881–51-0	− 0.61	P		L	B	B	61
	Diphenhydramine	58–73-1	3.27	P		L	T		5
	Fexofenadine	83799–24-0	2.81	POM		L	B	B	63
ANSTH/PYSTIM	Ketamine	6740–88-1	2.18	POM		L	T	T	16
ANSTH	Lidocaine	137–58-6	2.26	POM		G/L		B	13
ANTB	Azithromycin (WL)	83905–01-5	4.02	POM		L	T		3
	Erythromycin (WL)	114–07-8	3.06	POM		L	B		8
	Sulfamethoxazole (WL)	723–46-6	0.89	POM		L	T		5
	Sulfapyridine	144–83-2	0.35	POM		L	B	B	53
	Trimethoprim (WL)	738–70-5	0.91	POM		L	B	T	24
ANTD	Citalopram	59729–33-8	3.74	POM		L	B	B	55
	Maprotiline	10262–69-8	4.52	POM		L	T	T	8
	Mirtazapine	61337–67-5	3.03	POM		L	T	T	5
	Sertraline	79617–96-2	5.29	POM		L	T	T	8
	Venlafaxine (WL)	93413–69-5	3.28	POM		L	B	B	50
M (ANTD)	Norcitalopram	144025–14-9	3.53	M		L	I	T	8
	Norvenlafaxine	149289–30-5	3	POM		L		T	8
	O-Desmethylvenlafaxine (WL)	93413–62-8	2.72	M		L	B	B	61
BB	Acebutolol	37517–30-9	1.71	POM		L		T	5
	Atenolol	29122–68-7	0.16	POM		L	T	T	8
	Bisoprolol	66722–44-9	1.87	POM		L	B	T	24
	Celiprolol	56980–93-9	1.92	POM		L	T	T	11
	Propranolol	525–66-6	3.48	POM		L	B	T	26
BLAD	Trospium	10405–02-4	0.78	POM		L	T	T	5
BT	Clopidogrel	113665–84-2	3.82	POM		L	B	T	32
CAN	Bicalutamide	90357–06-5	2.8	POM		L	T	T	8
CHBP	Aliskiren	173334–57-1	2.45	POM		L	T	T	8
	Bethanidine	55–73-2	1.56	POM		L	T	T	18
	Disopyramide	3737–09-5	2.58	POM		L	T	T	18
	Flecainide	54143–55-4	3.78	POM		L	B	B	66
	Irbesartan	138402–11-6	5.31	POM		L	B	T	26
	Losartan	114798–26-4	4.01	POM		L	B	T	13
	Practolol	6673–35-4	0.79	POM		L	T		3
	Rivaroxaban	366789–02-8	2.18	POM		L	T	T	8
	Sotalol	3930–20-9	0.24	POM		L	T	B	21
	Telmisartan	144701–48-4	8.42	POM		L		T	13
M (CHBP)	Deacetyldiltiazem	42399–40-6	1.29	M		L	T	B	29
CHOL	Bezafibrate	41859–67-0	4.25	POM		L	T		8
CHOLIN	Carbachol	51–83-2	− 3.78	POM		L	I		3
CMED	Guaifenesin	93–14-1	1.39	GSL		L		T	3
	Methylephedrine	552–79-4	1.7	DD		L	T	T	8
CNS STIM	Caffeine	58–08-2	0.16	GSL		G/L	B	B	58
	Nikethamide	59–26-7	0.33	POM		L		T	3
M (CNS STIM)	Cotinine	486–56-6	0.34	M		L	T	T	13
DIAB	Metformin	657–24-9	− 1.4	POM		L		T	3
	Sitagliptin	486460–32-6	1.39	POM		L	T	T	5
M (DIUR)	Canrenone	976–71-6	2.68	M		L		T	3
EPD	Aminoglutethimide	125–84-8	0.82	POM		L		T	3
	Carbamazepine	298–46-4	2.25	POM		G/L	B	B	82
	Lamotrigine	84057–84-1	0.99	POM		L	B	B	74
	Oxcarbazepine	28721–07-5	1.11	POM		L	T	B	16
M (EPD)	10-Hydroxycarbamazepine	29331–92-8	1.11	M		L	T		3
	Carbamazepine-10.11-epoxide	36507–30-9		M		L	B	B	53
M (FLU)	Norephedrine (phenylpropanolamine)	14838–15-4	0.83	DD		L	T	B	18
FUNG	Fluconazole (WL)	86386–73-4	0.25	P		L	I	B	11
HOR	Boldenone	846–48-0	3.05	POM	C	L	T		3
LAX	Metoclopramide	364–62-5	2.62	POM		L	T	T	5
MALD	Proguanil	500–92-5	2.29	POM		L	B		18
MRELX	Methocarbamol	532–03-6	0.61	POM		L	I		8
NAU	Alizapride	59338–93-1	1.79	POM		L		I	5
	Cyclizine	82–92-8	2.97	POM		L	T	T	8
NSAID	Diclofenac	15307–86-5	4.02	POM		L	T	B	55
	Naproxen	22204–53-1	3.18	P		G/L	T		5
OPAN	Codeine	76–57-3	1.28	P/POM	B	L	B	B	29
	Dihydrocodeine	125–28-0	1.49	POM	B	L	T	T	13
	Hydrocodone	125–29-1	2.16	POM	A	L	B	B	34
	Meptazinol	54340–58-8	3.77	POM		L	T	T	8
	Methadone	76–99-3	3.93	POM	A	L	T		3
	Oxycodone	76–42-6	0.66	POM	A	L		T	3
	Pethidine	57–42-1	2.72	POM	A	L		T	3
	Tapentadol	175591–23-8	3.57	POM	A	L	B	B	16
M (OPAN)	Norcodeine	467–15-2	0.69	POM	B	L		T	3
PAR/FUNG	Ethylparaben	120–47-8	2.47	ING		G		T	3
PAR/PER	Propylparaben	94–13-3	3.04	ING		G		T	3
PERS	2-Methoxynaphthalene	93–04-9	3.47	ING		G		I	3
	Citral	5392–40-5	3.45	ING		G		I	3
	Isopropyl myristate	110–27-0	7.17	ING		G		B	45
	Isopropyl palmitate	142–91-6	8.16	ING		G	B		11
	Oxacyclohexadecan-2-one	106–02-5	5.8	ING		G		I	3
	p-Isopropyltoluene	99–87-6	4.1	ING		G		I	3
	Phenol	108–95-2	1.46	ING		G		B	11
	Vanillin	121–33-5	1.2	ING		G	B	B	45
PR	Ethenzamide	938–73-8	1.2	POM		L		I	3
M (PR)	4-Acetamidoantipyrine (4-AAA)	83–15-8	− 0.13	M		L		T	3
	4-Formylaminoantipyrine (4-FAA)	1672–58-8	0.5	M		L	T	T	5
	N-Desmethylpropafenone	86383–21-3	1.9	M		L		T	3
	N-Desmethyltapentadol	1300037–83-5	3.1	M		L	T	T	13
	Norcocaine	18717–72-1	1.96	M	A	L	T		3
	O-Desmethylnortramadol	na	2.3	M		L		B	13
	O-Desmethyltramadol	73986–53-5	2.3	M		L		B	18
PYSM	Amisulpride	71675–85-9	1.11	POM		L	B	B	47
	Amitriptyline	50–48-6	4.92	POM		L	T		3
PYSM/SED/T	Sulpiride	15676–16-1	0.57	POM		L	B	B	37
PYSTIM	2 C-D	24333–19-5	2.05	DD	A	L		T	3
	BDB	42542–07-4	2.15	DD	A	L		T	3
	Bufotenin	487–93-4	1.46	DD	A	L	I		3
PYSTIM/CNS STIM	Cocaine	50–36-2	2.3	POM/DD	A	L		T	3
PYSTIM	Methoxetamine	1239943–76-0	2.03	DD	B	L	T		3
	Methoxyphenamine	93–30-1	2.6	DD	A	L		T	3
	Pentedrone	879722–57-3	2.5	DD	B	L		I	3
PYSTIM/ANTD	Pipradrol	467–60-7	3.45	DD	C	L		T	5
M (PYSTIM)	Benzoylecgonine	519–09-5	− 1.32	POM	A	L	T		3
RETRO	Harman	486–84-0	2.65	POM		L		T	3
SED/T	Temazepam	846–50-4	2.19	POM	C	L	T		5
SKIN	Benzyl benzoate	120–51-4	3.97	GSL		G		T	3
	Nicotinamide	98–92-0	− 0.37	POM		L	B	T	13
ULC	Esomeprazole	119141–88-7	2.23	POM		L	T		3
	Ranitidine	66357–35-5	0.27	GSL		L	T		11
UV FILTER	2-Ethylhexyl salicylate	118–60-5	6.36	GSL		G	I	B	39
	Benzophenone-3	131–57-7	3.79	ING		G		B	8
	Benzyl salicylate	118–58-1	4.31	ING		G		B	8
	Homosalate	118–56-9	5	ING		G		I	3
	2-Ethylhexyl 4-methoxycinnamate	5466–77-3	5.3	ING		G	B	T	16

**Table 2:** List of plant protection products (together with Chemical Abstracts Number (CAS) and Log *K*_*ow*_) detected and their frequency (%) in extracts from the spot water samples collected from the River Itchen and Test in March and June. Code: *Fung* fungicide, *Herb* herbicide, *Ins* insecticide, *PGR* plant growth regulator, *TP* transformation product. Analytical detection: GC/MS = G; LC/MS = L. River detection: River Itchen = I; River Test = T; B = both rivers. PS = priority substance; WL = Watch List

Code	Compound	CAS	Log *K*_*ow*_	Detection by GC/MS or LC/MS	River detected in March	River detected in June	Detection (%) in all samples (*n* = 38)
Fung	Azaconazole ^2^	60207–31-0	2.32	G		B	11
	Azoxystrobin ^1^	(131860–33-8)	2.5	G & L	B	T	37
	Benomyl (decomposed to carbendazim) ^2^	(17804–35-2)	2.12	L		T	3
	Biphenyl ^2^	92–52-4	4.01	G		T	3
	Boscalid ^1^	188425–85-6	2.96	G/L	B	T	45
	Carbendazim ^2^	10605–21-7	1.52	L		T	3
	Cyproconazole ^1^	(94361–06-5)	2.9	L	T		3
	Epoxiconazole ^2^	(133855–98-8)	3.44	L	B		13
	Fenpiclonil ^2^	74738–17-3	3.86	G	I		3
	Fluopyram ^1^	(658066–35-4)	4.78	L	I		3
	Flusilazole ^2^	(85509–19-9)	3.7	L	T		3
	Flutriafol ^1^	(76674–21-0)	2.29	L	I		3
	Fluxapyroxad ^1^	(907204–31-3)	3.08	L	B		8
	Griseofulvin ^2^	126–07-8	− 0.31	L	B	T	32
	Mandipropamid ^1^	(374726–62-2)	3.2	L	I	T	8
	Metalaxyl ^3^	57837–19-1	1.75	G/L		I	3
	Penthiopyrad ^1^	(183675–82-3)	4.62	L	I		8
	Propamocarb ^1^	24579–73-5	0.84	G/L	B	I	8
	Propiconazole ^2^	(60207–90-1)	3.72	L	T		5
	Pyraclostrobin ^1^	(175013–18-0)	3.99	L	I		3
	Rabenzazole ^1^	(40341–04-6)	1.56	L	I		3
	Spiroxamine ^1^	(118134–30-8)	2.79	L	B		21
	Sulphur (S8) ^1^	10544–50-0	3.5	G	I		5
	Tebuconazole (WL) ^1^	(107534–96-3)	3.7	G/L	B		5
	Trifloxystrobin ^1^	(141517–21-7)	4.5	L	I		3
Herb	Atrazine ^2^ (PS)	(1912–24-9)	2.61	G/L	B	B	100
	Barban ^2^	101–27-9	3.41	G	T		3
	Benzoylprop-ethyl ^2^	22212–55-1	4.1	G	I		3
	Carbetamide ^1^	16118–49-3	1.78	G/L	I		3
	Chlorotoluron ^1^	(15545–48-9)	2.41	L	B		13
	Desmetryn ^2^	1014–69-3	2.38	G		T	3
	Diflufenican ^1^	(83164–33-4)	4.2	G/L	T		3
	Dimethenamid ^2^	(87674–68-8)	1.89	L	T		3
	Diuron ^1^ (PS)	(330–54-1)	2.68	L	B	B	63
	Flufenacet ^1^	(142459–58-3)	3.2	G/L	B		24
	Flurtamone ^2^	(96525–23-4)	2.8	L	T		3
	Isoproturon ^2^ (PS)	34123–59-6	2.5	L		T	3
	Isoxaben ^1^	(82558–50-7)	3.94	L	T		3
	Lenacil ^1^	(2164–08-1)	3.09	L	I		3
	Metazachlor ^1^	(67129–08-2)	2.49	G/L	B	T	26
	Monuron ^2^	(150–68-5)	1.94	L	T		8
	Prometryn (Caparol) ^2^	(7287–19-6)	3.51	L	T	T	3
	Propazine ^2^	(139–40-2)	2.93	L	T	T	11
	Propyzamide (Pronamide) ^1^	(23950–58-5)	3.43	G/L	B		13
	Sebuthylazine ^2^	(7286–69-3)	3.31	L	B		21
	Simazine ^2^ (PS)	(122–34-9)	2.18	L	B	B	100
	Simetryn ^2^	(1014–70-6)	2.8	L	T		3
	Terbucarb^2^	(1918–11-2)	5.2	G		I	3
	Terbuthylazine ^1^	(5915–41-3)	3.4	L	B		18
	Terbutryn (Terbutryne)(PS) ^2^	(886–50-0)	3.74	G/L	T	T	13
	Thidiazuron ^2^	(51707–55-2)	1.77	L	T		3
Insect	3,4,5-Trimethacarb ^2^	2686–99-9	2.55	G		I	3
	Acetamiprid (WL)^1^	135410–20-7	0.8	L	T	T	8
	Azobenzene ^2^	103–33-3	3.82	G	B		11
	Chlorantraniliprole ^1^	(500008–45-7)	2.6	L	I		5
	Clothianidin (WL) ^3^	(210880–92-5)	0.7	L	B		18
	d-Limonene ^2^	5989–27-5	4.57	G		B	18
	DEET (Diethyltoluamide) ^2^	(134–62-3)	2.02	G/L	B	B	76
	Dimethyl phthalate ^2^	131–11-3	2.47	G/L		I	3
	Fipronil ^2^	(120068–37-3)	4	L	T		3
	Imidacloprid (WL) ^3^	138261–41-3	0.57	L	B	B	68
	Methoprene ^2^	(40596–69-8)	5.5	L	B	B	37
	Metolcarb ^2^	(1129–41-5)	1.7	L	B		8
	Phosmet ^3^	732–11-6	2.95	G	I	I	5
	Pyrethrin ^1^	(121–21-1)	5.9	L		B	21
	Pyrethrins: Jasmolin ^1^	(4466–14-2)	5.4	L		I	3
	Trimethacarb (2.3.5-) ^2^	(12407–86-2)	2.66	L	B		26
	Vamidothion ^2^	(2275–23-2)	0.16	L		B	5
PGR	Ancymidol ^2^	(12771–68-5)	1.99	L	B		5
	Cyclanilide ^2^	(113136–77-9)	3.25	L	T	T	5
TP	1(3H)-Isobenzofuranone ^2^	87–41-2	3.02	G	B		26
	Atrazine 2-hydroxy ^2^	(2163–68-0)	2.61	L	B	T	8
	Atrazine-desethyl ^2^	(6190–65-4)	1.51	L	B	B	100
	Atrazine-desisopropyl ^2^	(1007–28-9)	1.15	L	B	B	87
	Dichlorobenzamide ^2^	(2008–58-4)	3.43	L	B	T	42
	Melamine ^2^	(108–78-1)	− 1.37	L	T	B	37

^1^Approved for use in the EU and UK

^2^Not approved for use in the EU or UK

^3^Approved for use in the EU but not approved for use in the UK. Griseofulvin is also used as a PPCP

## Results and discussion

Compounds found in the blanks were removed from the GC/MS target list. Analytes found in the LC/MS mobile phase were removed from the target list. Analytes present in the laboratory blank were also removed if their response was less than three times those of the extracted water sample. Following filtering a final list of identified analytes was compiled. Chemicals detected were classified into three groups, either pharmaceuticals and personal care products, plant protection products or industrial chemicals.

### Pharmaceuticals and personal care products identified by suspect screening

In both rivers, a total of 115 PPCPs were identified in March and June (Table 1); these included several compounds that have been or are currently on the WL of the EU WFD (2-ethylhexyl 4-methoxycinnamate, O-desmethylvenlafaxine, azithromycin, diclofenac, erythromycin, fluconazole, sulfamethoxazole, trimethoprim, venlafaxine) (European Commission 2020; Gomez Cortes et al. 2020; Loos et al. 2018; Carvalho et al. 2015). The proportion of categories detected were prescription only medication (60%), metabolite (12%), ingredient (12%), designer drug (9%), general sales list (4%) and only available from a pharmacy (3%). The pharmaceuticals found were subdivided according to their therapeutic use. It should be noted that the list of PPCPs is not comprehensive as their will be substances not amenable to extraction by the HLB-L sorbent disk. Some highly polar PPCPs were unlikely to be detected by the instrumental methods used. The log *K*_*ow*_ of the PPCPs ranged from − 3.78 to 8.42. The more non-polar chemicals were assigned as personal care products (e.g. UV filters) and were detectable by the GC/MS technique.

The presence of PPCPs in surface waters has been studied extensively using a range of analytical detection systems (Castro et al. 2021; Wilkinson et al. 2019). A similar approach to ours (HLB disk and LC/MS screening) has been used previously to detect PPCPs in surface waters (Gravell et al. 2020; Rimayi et al. 2019). These workers detected a similar wide range of substances to those found in our study.

### Plant protection products identified by suspect screening

In both rivers, a total of 81 PPPs were identified in March and June; these included those approved/not approved for use in the EU or UK and their transformation products (TPs) (Table 2). The three major classes of detected PPPs were fungicides (25 compounds (32%)), herbicides (26 compounds (33%)) and insecticides (19 compounds (24%)). Other types of compounds detected included transformation products (6 compounds) and plant growth regulators (2 compounds). Several compounds identified were classified as priority substances (atrazine, diuron, isoproturon, simazine and terbutryn) or are on the Watch List (acetamiprid, clothianidin, imidacloprid and tebuconazole) of the EU WFD. The list of compounds in Table 2 is not fully comprehensive as there will be compounds that are not extracted by the HLB-L sorbent disk and/or not detected by the instrumental methods used. This would include, for example, acid herbicides, glyphosate and metaldehyde. The log *K*_*ow*_ of the PPPs ranged from − 1.37 to 5.9. This range was similar to Ahrens et al. (2015) and Taylor et al. (2020 and 2021) who both used HLB sorbent contained in a passive sampling device.

Using a similar analytical approach to detect PPPs in two rivers in South East England, Taylor et al. (2020 and 2021) identified 111 pesticides, including 37 herbicides (33%), 36 fungicides (32%) and 22 insecticides (20%) in the River Arun and 128 pesticides, including 39 herbicides (30.5%)), 34 fungicides (26.5%) and 27 insecticides (21.0%) in the River Rother. Their data agree with our findings for the River Itchen and Test. This was not unexpected as the catchments are geographically close and have similar agricultural practices within these catchments. Pesticide screening in streams throughout Europe also found a broadly similar proportion of the three major classes of pesticides (herbicides (45%), fungicides (34%) and insecticides (21%)) (Casado et al. 2019).

Other studies in more diverse (non-chalk stream) catchments gave differing results. For example, in South Africa (The Hennops and Jukskei Rivers lying in the Hartbeespoort Dam catchment around Johannesburg), Rimayi et al. (2019) found that fungicides accounted for 37% of total pesticides; the same as in our study. The percentage of both insecticides and herbicides (18%) was different to our findings. This may be partly attributed to the peri-urban characteristics of this catchment. Moschet et al. (2014) sampled in five Swiss rivers (March–July) containing large areas of diverse crops and urban settlements within the respective catchments. They detected 104 parent compounds of which 52% were herbicides, higher than in our study probably relating to agricultural practices within the catchments. Interestingly they found an additional 40 transformation products, far higher than found in our study. This may be due to the differences in the analytical techniques used. A higher percentage of herbicides (81%) compared to our study was found by Pitarch et al. (2016) in surface waters downstream of a solid-waste treatment plant in Castellón, Spain. Here, the PPPs percentage was dominated by one herbicide, triazine. This may be due to the surrounding catchment being dominated by WWTP input rather than agricultural practices. As is evident from the above studies, the relative amounts of PPPs present are highly dependent on the land use within the catchment being monitored.

### Industrial chemicals identified by suspect screening

Thirty-five IND were identified in both rivers in March and June (Table 3). As PAHs are ubiquitous, persistent environmental pollutants were the major class of IND detected (14 compounds (40%)). The sources of these are expected to be derived from combustion and contamination from petroleum-based fuels along with tyre fragments. PAHs can be transported into the surface water via runoff from roads. Two of the PAHs (fluoranthene and indeno[1,2,3-cd] pyrene) are on the priority substances list of the EU WFD (Directive 2008/105/EC). Other types of compounds detected included unclassified industrial compounds (9 compounds) being mainly phenolic substances and solvents (5 compounds). Two polychlorinated biphenyls (PCBs) (2,2′,4,5,5′-Pentachloro-1,1′-biphenyl and 2,2′,5,5′-Tetrachloro-1,1′-biphenyl) were also found. This range of PAHs and PCBs are commonly detected in surface waters in the UK. Emelogu et al. (2013) found a similar range of these two classes of compounds at three sites on two streams of an agricultural catchment area in North East Scotland, UK. All the IND were detected using GC/MS.

**Table 3:** List of industrial chemicals (together with Chemical Abstracts Number (CAS) and Log *K*_*ow*_) detected and their frequency (%) in extracts from the spot water samples collected from the River Itchen and Test in March and June. Code: *ADH* adhesive, *FR* flame retardant, *IND* industrial usage, *ORG* organic use, *PAH* polycyclic aromatic hydrocarbon, *PCB* polychlorinated biphenyls, *PLAST* plasticiser, *SOLV* solvent. Analytical detection: GC/MS = G. River detection: River Itchen = I; River Test = T; B = both rivers. PS = priority substance

Code	Compound	CAS	Log *K*_*ow*_	Detection by GC/MS or LC/MS	River detected in March	River detected in June	Detection (%) in all samples (*n* = 38)
ADH	2,4,7,9-Tetramethyl-5-decyne-4,7-diol	126–86-3	3.11	G	B	T	37
FR	Triphenyl phosphate	115–86-6	4.59	G		B	13
IND	2-Ethylhexanoic acid	149–57-5	2.64	G	B		50
	2-Nitrophenol	88–75-5	1.79	G		T	3
	2,4,6-Tribromophenol	118–79-6	4.13	G	B	T	21
	3,5-Dimethylphenol	108–68-9	2.35	G		B	26
	Cinnamal (Cinnamaldehyde)	104–55-2	1.9	G		I	3
	Diethylene glycol	111–46-6	− 1.47	G	I		5
	p-Cresol (4-methylphenol)	106–44-5	1.94	G		B	8
	p-Isopropyltoluene (P-Cymene)	99–87-6	4.1	G		I	3
	Styrene	100–42-5	2.95	G		B	11
ORG	4-Octyne	1942–45-6	3.4	G		B	16
PAH	2-Methylnaphthalene	91–57-6	3.86	G	I	B	39
	Acenaphthene	83–32-9	3.92	G		B	8
	Benz[a]anthracene	56–55-3	5.76	G		T	5
	Dibenzofuran	132–64-9	4.12	G		T	3
	Dibenzothiophene	132–65-0	4.38	G		T	5
	Ethylbenzene	100–41-4	3.15	G		B	11
	Fluoranthene (PS)	206–44-0	5.16	G	T	B	50
	Fluorene	86–73-7	4.18	G		B	16
	Indeno[1,2,3-cd] pyrene (PS)	193–39-5	6.7	G		T	3
	Isopropylbenzene (Cumene)	98–82-8	3.66	G		T	3
	Methyl methanesulfonate	66–27-3	0.7	G		T	3
	Naphthalene (PS)	91–20-3	3.3	G		B	29
	n-Propylbenzene	103–65-1	3.69	G		I	5
	Perylene	198–55-0	6.3	G		T	3
	Pyrene	129–00-0	4.88	G		B	32
PCB	2,2′,4,5,5′-Pentachloro-1,1′-biphenyl	37680–73-2	5.68	G		B	5
	2,2′,5,5′-Tetrachloro-1,1′-biphenyl	35693–99-3	6.09	G		B	16
PLAST	Benzenesulfonamide, N-butyl	3622–84-2	2.1	G	B	B	92
	Bisphenol A	80–05-7	3.32	G	B		13
SOLV	Benzonitrile	100–47-0	1.56	G		T	3
	Bromoform	75–25-2	2.4	G		B	11
	Decahydronaphthalene (cis)	493–01-6	4.6	G	I		3
	Diphenyl sulfone	127–63-9	2.4	G	I	T	5
	p-Xylene	106–42-3	3.15	G		B	11

### Rainfall and flow data

During the week prior to sampling on the 7th of March 2019, there were a number of rainfall events with up to 9 mm of precipitation recorded per day. Prior to the second sample collection on the 20th of June 2019, there were a number of rainfall events (07/06–12/06) with precipitation ranging from 9 to 22 mm. This was followed by a period of dryness (13/06–17/06) with precipitation on the 18th (5.5 mm) and 19th of June (3.7 mm). There was no rainfall on the day of the sample collection.

As the two rivers are chalk streams and are predominately ground water fed, they have a baseflow regime. Hence, their overall flow regime is not significantly affected by heavy precipitation. However, pollutants can enter the river via subsequent run off events within the catchment following periods of heavy rain. Flow rates for both rivers were higher in March than in June. Data also showed that the rivers experienced a small increase in flow after the above-mentioned precipitation episodes.

### Spatial and temporal occurrence of organic chemicals

#### PPCPs

**Fig. 2:**
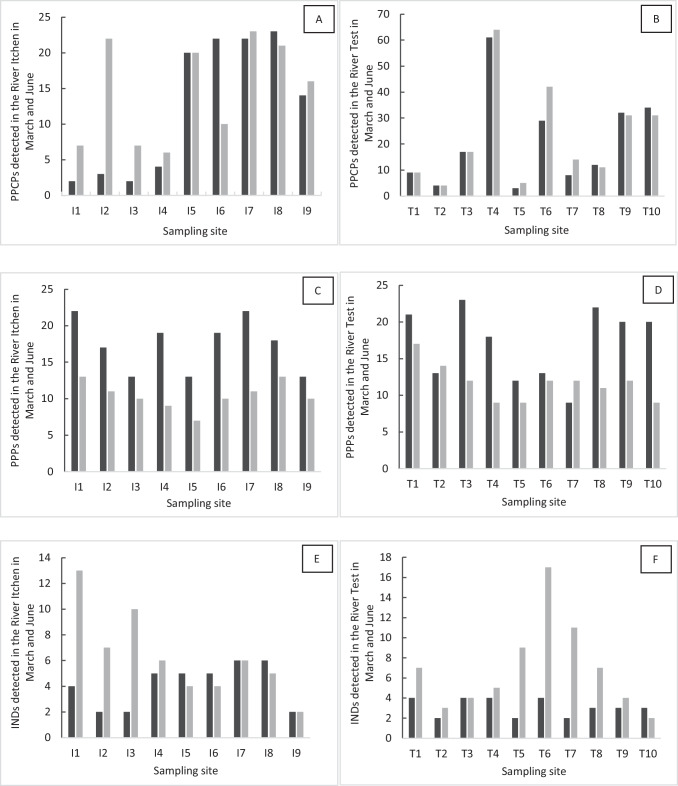
The number of compounds detected at each site on the River Itchen (I1–I9) (panels **A**, **C** and **E**) and River Test (T1–T10) (panels **B**, **D** and **F**) on the 7th of March 2019 (black bars) and on the 20th of June 2019 (grey bars). **A** Pharmaceuticals and personal care products (PPCPs) detected in the River Itchen; **B** pharmaceuticals and personal care products (PPCPs) detected in the River Test; **C** plant protection products (PPPs) detected in the River Itchen; **D** plant protection products (PPPs) detected in the River Test; **E** industrial chemicals (IND) detected in the River Itchen; **F** industrial chemicals (IND) detected in the River Test. Waste water treatment plants are located near I2, I5 and I6 on the River Itchen and near T1, T3, T4, T6, T7, T8, T9 and T10 on the River Test. Sites I1, I2 and I3 are tributaries of the main River Itchen, and sites T2, T3, T5, T7 and T8 are tributaries of the main River Test

In the River Itchen (Fig. 2A), PPCPs had a similar number of total detections in both March (110 substances) and June (132 substances). PPCPs showed a similar number of detections at each site in both March and June apart from site I2. Here, there was an order of magnitude more detections in June (22 substances) compared to March (2 substances) (Fig. 2A). One of the reasons for more detections at this site and at sites I1, I3 and I4 could be inputs from CSOs and leakage arising from septic tanks due to the high rainfall events preceding sample collection. As these site locations are rural, there is a higher density of septic tanks compared to sites further down the river that have mains sewerage connection. Discharges from CSOs have been found to be a major source of emerging contaminants into river catchments (Petrie 2021) contributing up to 90% of annual loads (Phillips et al. 2012). In contrast, Munro et al. (2019) found no correlation of increase in PPCP detections with CSO events; however, this was for a heavily urbanised tidal river catchment. From site I5 and onwards, there were more PPCPs detected with the likely source being the number of WWTP discharges (Kasprzyk-Hordern et al. 2009) in this stretch of the river (Table S1). The decrease in PPCPs detected at site I9 for both months (March 14 substances; June 16 substances) is likely due to attenuation due to dilution effects as the Itchen Navigation joins the main river at this point. The maximum number of compounds detected at any of the sampling sites (I1–I9) was 23.

PPCPs in the River Test (Fig. 2B) were spatially highly variable due to difference in hydrology, wastewater management and urbanisation. Such variability has been observed in other river systems in the UK (Burns et al. 2018). Seasonally, the PPCPs showed a similar pattern in terms of total number of detections in both March (209 substances) and June (228 substances). Overall, compared to the River Itchen (March (110 substances) and June (132 substances), the number of compounds detected was higher, indicating a larger pollution load in this river. The maximum number of detections (62 substances) was at site T4. This is where effluent directly discharges from the largest WWTP (population equivalent ~ 63,000) on the River Test within our study area (Table S3). As expected there were far fewer compounds detected (Fig. 2B) on the tributary river sites (T2, T3, T5, T7 and T8—Table S2) compared to the sites (T1, T4, T6, T9 and T10—Table S2) on the main river. All the compounds detected at site T1 were subsequently detected downstream at all the other sampling sites on the main river. At tributary sites which receive WWTP inputs (T3 and T8), PPCPs are detected for the first time and are then always detected at the main river sites downstream. At the other tributary sites, there were some PPCPs detected that did not appear in the subsequent downstream main river sites due to attenuation effects. Tributaries have been shown to contribute little to the overall load of pollutants in river systems but can have a different chemical profile (Müller et al. 2020).

#### PPPs

For both rivers (Fig. 2C and D), the pattern of PPP detections was similar with a higher number of detections always being found in March. In the River Itchen, the total number of detections in March was 156 substances and in June was 86 substances. In comparison, in the River Test, the total number of detections in March was 171 substances and in June was 110 substances. The maximum number of detections (~ 20 compounds) in both rivers was similar. This observation reflects the increased use of PPPs in the spring growing season. For example, herbicides are applied widely to control broad-leaved weeds in grassland and some cereal crops during the period of March to May (Townsend et al. 2018). This increased use of PPPs and in particularly herbicides has also been reported by Taylor et al. (2021) in South East England. Unlike with the PPCPs, there is no generalised increase in the number of detections at the downstream sampling points. This is likely to be due to the continual run off of PPPs from agricultural activities across the catchment. However, the make-up of the individual PPPs found at each site was different reflecting differences in local land use and associated agricultural practices. Herbicides (43% March and 36% June) were the main product used in the catchments, followed by fungicides (33% March and 28% June). There was a notable change in the percentage use of insecticides in the warmer summer months (19% March and 31% June) when insects are more prevalent. Some of the PPPs were only detected in either March or June, and some were unique for a particular site. This again reflects the changes in seasonal agricultural practice within the catchment, i.e. herbicide use decreases during the warmer summer months (Townsend et al. 2018).

#### IND

For both rivers (Fig. 2E and F), there was an increase of IND detections in June. In the River Itchen (Fig. 2E), the total number of detections increased from 37 to 57, and in the River Test, the increase was from 31 to 69. For these volatile and semi-volatile compounds, the warmer weather may lead to their increased solubility and mobility within the environment. There was no discernible pattern of the distribution of IND compounds down the two river catchments. The most common type of compounds found in March were plasticisers detected in 35% of samples. In June this changed to PAHs being detected in 56% of samples with plasticisers reducing to 13%.

### Detection frequency of PPCPs and PPPs

**Fig. 3:**
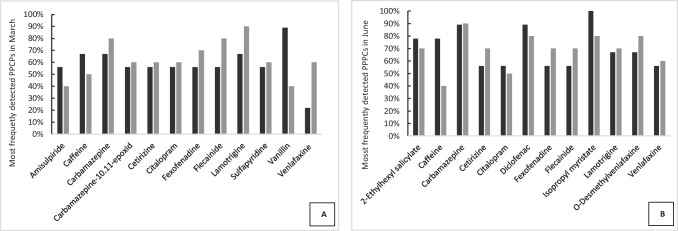
The twelve most frequently detected pharmaceuticals and personal care products (PPCPs) found in the River Itchen (black bar) and River Test (grey bar) on the 7th of March 2019 (**A**) and on the 20th June 2019 (**B**). Criterion used for selection was the compound that was detected in both rivers above a threshold of greater than 50% in one of the rivers

**Fig. 4:**
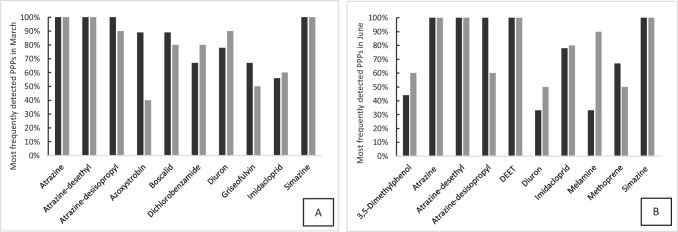
The ten most frequently detected plant protection products (PPPs) found in the River Itchen (black bar) and River Test (grey bar) on the 7th of March 2019 (**A**) and on the 20th of June 2019 (**B**). Criterion used for selection was the compound that was detected in both rivers above a threshold of greater than 50% in one and 30% in the other

The frequency of the compounds detected in the River Itchen and the River Test in March and June (Tables 1, 2 and 3) was determined to elucidate the most common pollutants present during these time periods. The most frequently detected PPCPs and PPPs for these two months is presented in Figs. 3 and 4 and discussed further below.

#### PPCPs

Of the sixteen most frequently detected compounds, eight (caffeine, carbamazepine, cetirizine, citalopram, fexofenadine, flecainide, lamotrigine, venlafaxine) of these were common to both months (Fig. 3A and B). All of these compounds have been reported to be present in UK rivers (Wilkinson et al. 2019; Kasprzyk-Hordern et al. 2008). The other four (amisulpride, carbamazepine-10.11-epoxide, sulfapyridine, vanillin) compounds found in March were also detected in June but not with a high enough frequency to be included in the top twelve analytes. Of the four compounds detected in June, o-desmethylvenlafaxine (3rd Watch List compound under the WFD) was also detected in March in both rivers but not at a high frequency. Diclofenac and 2-ethylhexyl salicylate were only detected in one river in March, whilst isopropyl myristate was not detected in March.

#### PPPs

Of the fourteen most frequently detected compounds, six (atrazine, atrazine-desethyl, atrazine-desisopropyl, diuron, imidacloprid, simazine) of these were common to both months (Fig. 4A and B). Atrazine, diuron and simazine are legacy pesticides as they have no UK or EU approval for their use. It is expected that these compounds have contaminated the groundwater that is abstracted for agricultural use within these river catchments. These compounds are frequently detected in European rivers. Poulier et al. (2015) detected atrazine between 60 and 100% in water samples collected from French rivers. Taylor et al. (2020) reported lower detection frequencies for atrazine (12%) and simazine (8%) but a higher detection frequency for diuron (99%) in a river catchment in the south east of England. In addition, these PPPs have been detected (atrazine 25%; simazine 20%) in groundwater from chalk aquifers (Stuart et al. 2011). Two of the metabolites of atrazine had a similar detection frequency to the parent pesticide. This was different to Taylor et al. (2021) who reported lower occurrences (~ 30%) of the metabolites compared to the parent pesticide. Imidacloprid (an active ingredient in many veterinary flea products) was found in rivers in England (Perkins et al. 2021; Taylor et al. 2021) at a similar detection frequency to our study. Its agricultural use is now prohibited so WWTPs are expected to be the major source of this compound.

The other four compounds found in March were fungicides (azoxystrobin, boscalid, griseofulvin) and a metabolite (dichlorobenzamide) of the fungicide fluopicolide. These compounds were only detected in June in the River Test at much lower frequencies. Higher detections were expected in March due to their spring time application to control fungal infections. These findings are in broad agreement with Taylor et al. (2021). Of the four other most frequently detected compounds in June, three are used to control insects (DEET, methoprene, pyrethrins), and the other is a metabolite (melamine) of the insecticide cyromazine. It was expected that insecticides would be used more in the summer months compared to spring. Recently, DEET is considered an ubiquitous pollutant and is frequently found in surface and groundwaters (Marques dos Santos et al. 2019; Merel & Snyder 2016).

### Ecological impact of pollutants

Our qualitative suspect screening work showed that there was a cocktail of pollutants present in both rivers (Figs. S3 and S4). The analytical procedure used in this study, however, does not enable concentrations of individual pollutants to be estimated. Using this approach, we identified 115 PPCPs, 81 PPPs and 35 INDs. The ecological impact of this complex chemical mixture on chalk stream flora and fauna is unknown, particularly on macroinvertebrate biodiversity (Johnson 2019). Most toxicological studies are undertaken using a single compound tested against a single species, and there is no standardised system for exposure. For example, the herbicide atrazine (the most frequently detected PPP in our study) and its metabolites have been shown to inhibit photosynthesis (Graymore et al. 2001; Ralston-Hooper et al. 2009) and also act as an endocrine disruptor adversely affecting fish (Tillitt et al. 2010). Other PPPs detected with a high frequency have also been reported to have toxicological effects. The fungicides azoxystrobin and boscalid are considered toxic to algae, fish and freshwater invertebrates (Elskus 2014). The neonicotinoid insecticide imidacloprid has been reported to be toxic to macro-invertebrates in surface waters (Van Dijk et al. 2013).

Similarly, there have been a number of reports highlighting the effect of PPCPs on aquatic fauna. For example, Sumpter and Margiotta-Casaluci (2022) predicted that the concentration of 32 neuroactive pharmaceuticals in surface waters may be high enough to elicit pharmacological effects in wild fish. Fexofenadine detected in our study with a high frequency in both months (Fig. 3) can cause changes in the behaviour of the damsel fly (Zygoptera) (Jonsson et al. 2014). Likewise, caffeine and diclofenac, found at a high frequency in June in both rivers (Fig. 3B), have been shown to cause endocrine and reproductive changes in fish (Godoi et al. 2020). The antidepressant venlafaxine and its major metabolite o-desmethylvenlafaxine were frequently found in both rivers and have been shown to affect the behaviour and mortality of fish (Sehonova et al. 2018). Interestingly, more recent work has started to investigate the impact of chemical mixtures on freshwater ecosystems (Barber et al. 2022; Covert et al. 2020; Nowell et al. 2018).

## Conclusions

With a suspect screening approach using two chromatographic techniques, we were able to identify over 200 organic chemicals in spot water samples. Some of the chemicals detected are either priority substances or on the Watch List of the EU WFD. Others were classified as legacy pollutants that have no current approval for use in the UK. Hence, the chalk stream biota are exposed to a complex mixture of chemicals that can act either antagonistically or synergistically in the environment. The qualitative analytical approach used gives an indication of the pollutants present that could guide future monitoring campaigns and toxicological investigations and help direct mitigation strategies within the catchments. Future work could use passive sampling devices to obtain time-weighted average sampler extracts that could be used in effect-directed analysis investigations (Sonavane et al. 2018).

## Supplementary Information

Below is the link to the electronic supplementary material.

Supplementary file1 (DOCX 2422 KB)

## Data Availability

The full dataset in Excel format is available on request to the corresponding author.
