# Supplementary material for: Occurrence of organic pollutants in the River Itchen and River Test—two chalk streams in Southern England, UK
Source: Environ Sci Pollut Res Int. 2022 Oct 7;30(7):17965–83. doi: 10.1007/s11356-022-23476-w (PMC9928825; doi:10.1007/s11356-022-23476-w)
Supplement: Supplementary file 1 — Supplementary file1 (DOCX 2422 KB) [file 11356_2022_23476_MOESM1_ESM.docx]

**Electronic supplementary material**

**Occurrence of organic pollutants in the River Itchen and River Test - two chalk streams in southern England, UK**

Rosamund F. A. Robinson,^a^ Graham A. Mills,^b^ Anthony Gravell,^c^ Melanie Schumacher^c^ and Gary R. Fones,^a^*

^a^School of the Environment, Geography and Geosciences, University of Portsmouth, Burnaby Road, Portsmouth, PO1 3QL, UK.

^b^School of Pharmacy and Biomedical Sciences, University of Portsmouth, White Swan Road, Portsmouth, PO1 2DT, UK.

^c^Natural Resources Wales, Faraday Building, Swansea University, Singleton Campus, Swansea, SA2 8PP, UK.

*Corresponding author: Gary Fones: gary.fones@port.ac.uk; +44(0)23 9284 2252

**List of Figures**

**Figure S1.** Map of the River Itchen catchment area showing the positions of sampling sites (■), waste water treatment plants (▲), gauging stations (◇) and historic landfill sites ( ). The two insets show the location of the study site within southern England. Some features of this map are based on spatial data licences from the UK Centre for Ecology & Hydrology, UKCEH and contains Ordnance Survey data crown copyright and database right 2022, crown copyright and database rights 2021 OS 100025252.

**Figure S2.** Map of the River Test catchment area showing the positions of sampling sites (■), waste water treatment plants (▲), gauging stations (◇) and historic landfill sites ( ). The two insets show the location of the study site within southern England. Some features of this map are based on spatial data licences from the UK Centre for Ecology & Hydrology, UKCEH and contains Ordnance Survey data crown copyright and database right 2022, crown copyright and database rights 2021 OS 100025252.

**Figure S3:** Percentage of the different types of compounds detected in water samples collected on the 7 March 2019 in the River Itchen and River Test.

**Figure S4:** Percentage of the different types of compounds detected in water samples collected on the 20 June 2019 in the River Itchen and River Test.

**List of Tables**

**Table S1:** Sample site code and name with description and location details for the River Itchen catchment

**Table S2:** Sample site code and name with description and location details for the River Test catchment

**Table S3**: Location of wastewater treatment plants on the Rivers Itchen and Test (order of river flow)

**
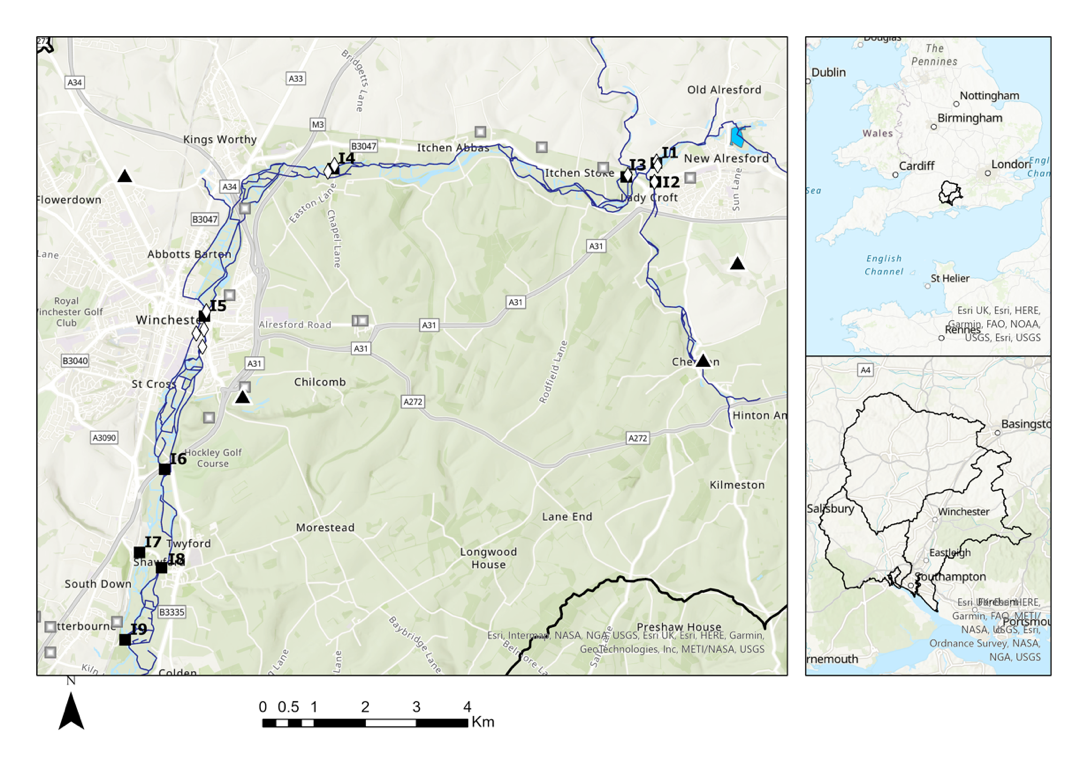
**

**Figure S1**: Map of the River Itchen catchment area showing the positions of sampling sites (■), waste water treatment plants (▲), gauging stations (◇) and historic landfill sites ( ). The two insets show the location of the study site within southern England. Some features of this map are based on spatial data licences from the UK Centre for Ecology & Hydrology, UKCEH and contains Ordnance Survey data crown copyright and database right 2022, crown copyright and database rights 2021 OS 100025252.

**
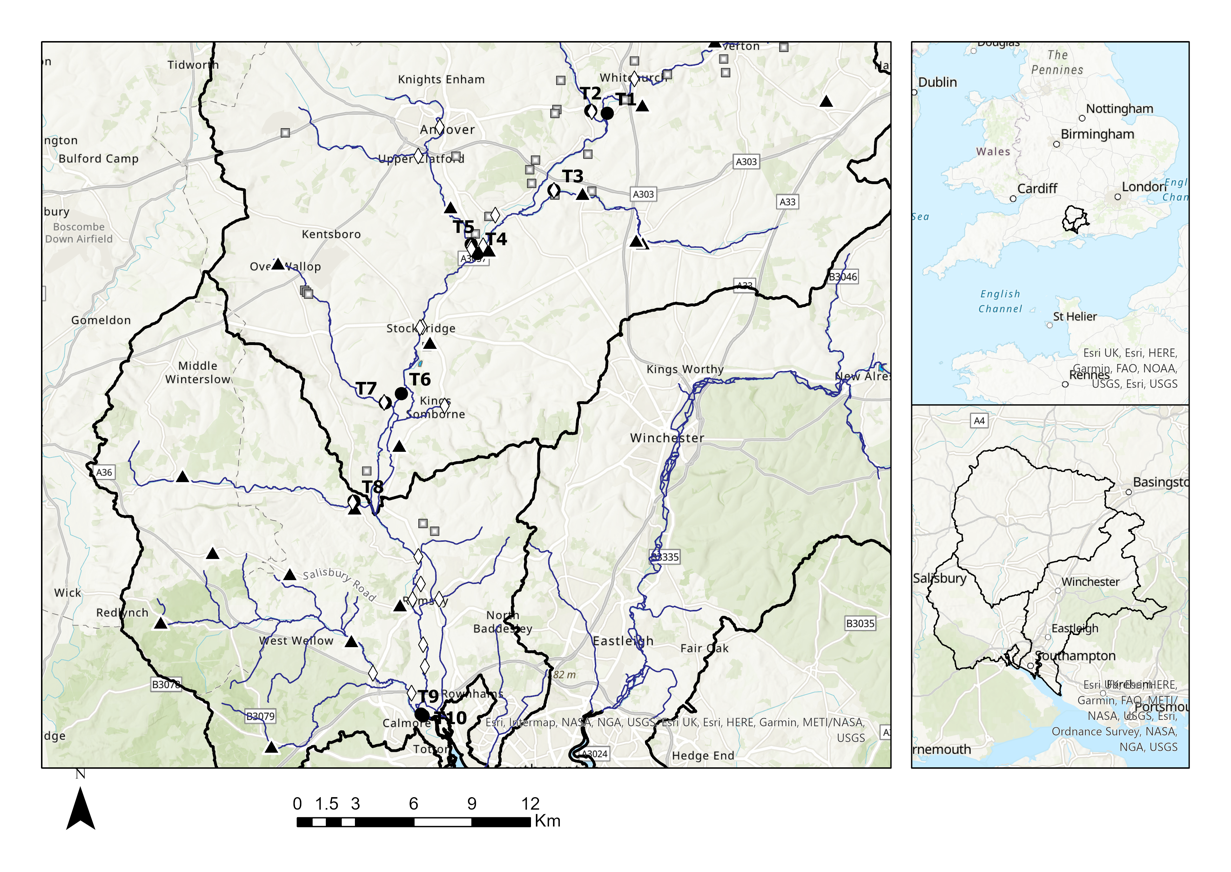
**

**Figure S2:** Map of the River Test catchment area showing the positions of sampling sites (■), waste water treatment plants (▲), gauging stations (◇) and historic landfill sites ( ). The two insets show the location of the study site within southern England. Some features of this map are based on spatial data licences from the UK Centre for Ecology & Hydrology, UKCEH and contains Ordnance Survey data crown copyright and database right 2022, crown copyright and database rights 2021 OS 100025252.

**Figure S3:** Percentage of the different types of compounds detected in water samples collected on the 7 March 2019 in the River Itchen and River Test.

Pharmaceuticals and personal care products (PPCPs): PYSM and PYSTIM: Psychotic medication and Psychotic stimulant; Heart: Medication for treating problems with the heart or circulation; CNS STIM: Central Nervous System Stimulant; EPD: Epileptic Drug; OPAN: Opioid based drug; PAIN: Pain relief; ANTB: Antibiotic; ANTD: Antidepressant; ANHIS: Antihistamine; NSAID: Non-steroidal Anti-Inflammatory Drugs; PERS: Personal care products; UV: UV filters; OTHER: Percentage of unclassified PPCPs.

Plant protection products (PPPs): HERB: Herbicide; FUNG: Fungicide; INSECT: Insecticide; LARV: Larvicide; PGR: Plant Growth Regulator.

Industrial Chemicals: ADH: Adhesive; PAH: Polycyclic aromatic hydrocarbons; PLAST: Plasticisers; SOLV: Solvents; IND: Percentage of unclassified industrial chemicals.

**Figure S4:** Percentage of the different types of compounds detected in water samples collected on the 20 June 2019 in the River Itchen and River Test.

Pharmaceuticals and personal care products (PPCPs):

PYSM and PYSTIM: Psychotic medication and Psychotic stimulant; Heart: Medication for treating problems with the heart or circulation; CNS STIM: Central Nervous System Stimulant; EPD: Epileptic Drug; OPAN: Opioid based drug; PAIN: Pain relief; ANTB: Antibiotic; ANTD: Antidepressant; ANHIS: Antihistamine; NSAID: Non-Steroidal Anti-Inflammatory Drugs; PERS: Personal care products; UV: UV filters; OTHER: Percentage of unclassified PPCPs.

Plant protection products (PPPs): HERB: Herbicide; FUNG: Fungicide; INSECT: Insecticide; LARV: Larvicide; PGR: Plant Growth Regulator.

Industrial Chemicals: PAH: Polycyclic aromatic hydrocarbons; PLAST: Plasticisers; SOLV: Solvents; PCB: Polychlorinated Biphenyls; FR: Flame retardants; IND: Percentage of unclassified industrial chemicals.

**Table S1:** Sample site code and name with description and location details for the River Itchen catchment

| **Site code** | **Site Name** | **Type** | **Description** | **Location**  **Eastings**  **Northings** | **Location**  **Latitude**  **Longitude** |
| --- | --- | --- | --- | --- | --- |
| I1 | River Alre | Tributary | Site is downstream of the headwaters of the River Alre near Bishop Sutton, and downstream of watercress beds and a fish farm. Rural properties discharge wastewater into river | E. 457440  N. 132590 | 51.0898  -1.1812 |
| I2 | Cheriton Stream at Winchester  Road | Tributary | Site is 0.2 km from housing estates at Woodland, New Alresford. It is downstream of the Cheriton WWTP, a fish farm and watercress beds | E. 457418  N. 132220 | 51.0864  -1.1816 |
| I3 | Candover Brook at Itchen Stoke | Tributary | Site is next to watercress beds and upstream before the join with the combined River Alre and Cheriton Stream | E. 456850  N. 132320 | 51.0874  -1.1897 |
| I4 | River Itchen at Easton Bridge | Main river | Site is 0.5 km downstream of a spring, and near some cottages at Martyr Worthy | E. 451120  N. 132480 | 51.0894  -1.2715 |
| I5 | River Itchen at Water Lane | Main river | Water Lane is in the centre of Winchester and 2 km downstream of industrial estates and a nature reserve. The Harestock WWTP discharges upstream of this site | E. 448609  N. 129591 | 51.0636  -1.3077 |
| I6 | River Itchen at Hockley Link | Main river | Site is under the M3 motorway 1 km downstream of a hospital and 2.5 km downstream of the WWTP at Morestead Road, this WWTP discharges to the strata. | E. 447830  N. 126590 | 51.0367  -1.3192 |
| I7 | Itchen Navigation Canal | Main river | Shawford House, the site is at the junction of the Itchen Navigation canal where it joins the River Itchen | E. 447340  N. 124960 | 51.0221  -1.3264 |
| I8 | River Itchen at Norris’s Bridge | Main river | Site is on a separate eastern channel of the River Itchen | E. 447770  N. 124660 | 51.0193  -1.3203 |
| I9 | River Itchen at Otterbourne | Main river | The confluence of the River Itchen and the Itchen Navigation canal at Otterbourne and east of a reservoir, west of boreholes. Extraction point for a pumping station. | E. 447060  N. 123250 | 51.0067  -1.3306 |

**Table S2:** Sample site code and name with description and location details for the River Test catchment

| **Site code** | **Site Name** | **Type** | **Description** | **Location**  **Eastings**  **Northings** | **Location**  **Latitude**  **Longitude** |
| --- | --- | --- | --- | --- | --- |
| T1 | River Test, Whitchurch | Main river | South west of Whitchurch approximately 2 km downstream from the WWTP although this discharges to groundwater, | E. 444893  N. 146203 | 51.2133  -1.3587 |
| T2 | Bourne Rivulet | Tributary | Site is 0.2 km downstream of Hurstbourne Priors and 0.8 km upstream of the junction with the main river | E. 444044  N. 146342 | 51.2146  -1.3708 |
| T3 | River Dever at Bransbury | Tributary | 1.2 km downstream of Bransbury, where a fish farm and a WWTP are located, the WWTP treats wastewater from Bransbury and St Mary Bourne and discharges to the river | E. 442150  N. 142240 | 51.1778  -1.3984 |
| T4 | River Test at Mayfly Fullerton | Main river | The site is adjacent to the effluent discharge pipe from Fullerton WWTP | E. 438201  N. 139000 | 51.1492  -1.4552 |
| T5 | River Anton at Fullerton | Tributary | The site is 0.5 km upstream from where this river joins the River Test and downstream of site T4 | E. 437880  N. 139460 | 51.1531  -1.4598 |
| T6 | River Test, Houghton | Main river | East of Houghton and 2.5 km downstream of Stockbridge WWTP | E. 434277  N. 131784 | 51.0844  -1.512 |
| T7 | Wallop Brook | Tributary | The site is 0.5 km upstream from where this river joins the River Test | E. 433461  N. 131316 | 51.0802  -1.5237 |
| T8 | River Dun at Dunbridge | Tributary | 2 km downstream of Lockerley and 1 km upstream from where this river joins the River Test. The site is close to the site of the Dunbridge WWTP which drains into the underlying strata | E. 431838  N. 126250 | 51.0347  -1.5473 |
| T9 | Testwood River Test | Main river | The site is 0.3 km downstream of a trout farm and Nursling mill, 0.5 km west of Testwood industrial and housing estates and 1 km east of Nursling. The M27 motorway is 2 km north. | E. 435300  N. 115300 | 50.9361  -1.499 |
| T10 | Testwood Lakes | Main river | The site is 0.2 km upstream of the main river flowing east of Little Testwood Lake before joining the main river this site is close to the water treatment works at Testwood | E. 435400  N. 115200 | 50.9352  -1.4976 |

**Table S3**: Location of wastewater treatment plants on the Rivers Itchen and Test (order of river flow)

**River Itchen**

| **Eastings** | **Northings** | **Discharge to Strata or River** | **Site Name** | **PE** |
| --- | --- | --- | --- | --- |
| 458350 | 128764 | Cheriton Stream | Cheriton | Not Known |
| 459021 | 130671 | Strata | New Alresford | 5354 |
| 447050 | 132382 | River Itchen | Harestock | 15774 |
| 449353 | 128051 | Strata | Morestead Road, Winchester | 36625 |
| 446613 | 118129 | River Itchen | Chickenhall, Eastleigh | 100830 |

**River Test**

| **Eastings** | **Northings** | **Discharge to Strata or River** | **Site Name** | **PE** |
| --- | --- | --- | --- | --- |
| 453959 | 154893 | Strata | Hannington | 38 |
| 456460 | 151092 | Strata | Ivy Down Lane, Oakley | 5051 |
| 456183 | 146942 | Strata | North Waltham | 816 |
| 450443 | 149985 | Strata | Overton | 4477 |
| 446703 | 146700 | Strata | Whitchurch | 4757 |
| 441478 | 157571 | Strata | Barn Close, Ashmansworth | 20 |
| 446726 | 139597 | River Dever | East Gratton | 367 |
| 446385 | 139737 | River Dever | Saddler Close, Sutton Scotney | 51 |
| 443622 | 142150 | River Dever | Barton Stacey | 3583 |
| 438799 | 139229 | River Test | Chilbolton | 1155 |
| 425710 | 150417 | Strata | Ludgershall | 4143 |
| 436817 | 141459 | River Test | Fullerton | 62813 |
| 435753 | 134485 | River Test | Stockbridge | 741 |
| 427923 | 138562 | Wallop Brook | Evans Close, Over Wallop | 332 |
| 434172 | 129188 | River Test | Kings Somborne | 2012 |
| 423001 | 127621 | River Dun | East Grimstead | 2968 |
| 431873 | 125957 | Strata | Dunbridge | 106 |
| 434212 | 120974 | River Test | Romsey | 18871 |
| 428531 | 122577 | River Blackwater | Graemar Cottages | 88 |
| 421876 | 120082 | River Blackwater | Redlynch | 795 |
| 431703 | 119136 | River Blackwater | West Wellow | 4341 |
| 424553 | 123674 | River Blackwater | Whiteparish | 1101 |
| 427583 | 113695 | River Cadnam | Canterton Lane Brook | 41 |

PE: Population equivalent is a quantity measure used to represent how much sewage the treatment facility needs to treat. It consists of the calculated equivalent number of people who are likely to contribute to the amount of sewage in the catchment.

**Data Sources:**

Southern Water (2020). Drainage and Wastewater Management Plan - Test and Itchen Catchment <https://www.southernwater.co.uk/media/3908/test-and-itchen-dwmp-strategic-context.pdf>.

The Rivers Trust (2022) Is your river fit to play in?

<https://theriverstrust.org/key-issues/sewage-in-rivers>
